# The prognostic and diagnostic value of tissue inhibitor of metalloproteinases gene family and potential function in gastric cancer

**DOI:** 10.7150/jca.57808

**Published:** 2021-05-13

**Authors:** Zhao Li, Qinwen Jing, Liucheng Wu, Jiansi Chen, Mingwei Huang, Yuzhou Qin, Tingan Wang

**Affiliations:** Department of Gastrointestinal Surgery, Guangxi Medical University Cancer Hospital, Guangxi Clinical Research Center for Colorectal Cancer, Nanning 530021, Guangxi Zhuang Autonomous Region, China.

**Keywords:** gastric cancer, TIMP, prognosis, diagnosis, biomarker.

## Abstract

**Background:** Tissue inhibitor of metalloproteinases (TIMP) gene family, including *TIMP1*, *TIMP2*, *TIMP3* and *TIMP4*, was found to be correlated with serval cancers. Still the diagnostic and prognostic study of it in gastric cancer (GC) have few reports.

**Methods and materials:** In this study, the gene expression and clinical data were acquired from the Cancer Gene Atlas (TCGA), function enrichment was used by several databases for verifying known function. Operating characteristic (ROC) curves with area under the curve (AUC) used to assess diagnostic value. Survival analysis and joint-effects survival analysis was performed by the Kaplan-Meier curve. The results were adjusted by cox-regression model. Nomogram is used to directly predict the survival rate for individual GC patient. The potential mechanism for diagnostic and prognostic value was assessed by gene set enrichment analysis (GSEA). Further functions of gene were verified by cell proliferation, migration and invasion assays in human gastric cancer cell line.

**Results:** *TIMP1* was expressed in GC tissue was higher than normal gastric tissue. *TIMP3* and *TIMP4* have expressed in normal gastric tissue were higher than GC tissue. *TIMP1*, *TIMP3* and *TIMP4* have potential diagnostic value (AUC=0.842, 0.729, 0.786 respectively; all P<0.01). Low expression of *TIMP2* and *TIMP3* associated with favorable overall survival (all P<0.05). *TIMP2* and *TIMP3*, which had significantly affection of prognosis were found having some function such as tRNA processing, cell cycle pathway ncRNA processing. The silencing of TIMP3 could inhibit the migration and invasion of gastric cancer cell.

**Conclusion:** We analyzed the TIMP gene family in GC, and the prognostic and diagnostic value. *TIMP1* and *TIMP2* could be used as diagnostic biomarkers in GC. *TIMP2* and *TIMP3* could be used as potential biomarkers for GC's prognosis.

## Introduction

Gastric cancer (GC) is the most common cancer type around world. In the United States, the estimated new GC cases are 27600, and the estimated death cases of GC is 11010 in 2020^1^. The Surveillance, Epidemiology and End Results Program (SEER, https://seer.cancer.gov, accessed in 1st July) indicates that GC takes 1.5% of all new cancer cases, and 5-year relative survival rate is only 32.0%. There are many biomarkers former researcher has been found, could be used for predicting the prognosis and diagnosis for GC 2. However there still don't have a golden biomarker standard for diagnosis and prognosis for GC patients, the most sensitive and widely used measure is pathological diagnosis.

Tissue inhibitor of metalloproteinases (TIMP) gene family, having four sub-members *TIMP1*, *TIMP2*, *TIMP3* and *TIMP4* has essential functions in the inhibition of matrix metalloproteinases (MMPs) and cell signaling such as cell death, proliferation and angiogenesis 3. Besides, those 4 genes were found to be correlated with many different types of cancer's occurrence 4, 5. The diagnostic and prognostic value and potential mechanism of individual *TIMPs* were illustrated in various cancer types such as TIMP1 in gastric cancer for prediction 6, *TIMP1* and *TIMP2* in colon cancer for prognosis 7, 8 and *TIMP4* in astrocytoma for diagnosis 9.

Since there are many studies about individual one of the *TIMPs*, there has no systematically research about prognostic and diagnostic value for whole *TIMP* family at mRNA level. The present study made a compensate, by the reliable open access data obtained from the Cancer Gene Atlas (TCGA), compared the differences expression level of every gene, and made survival and functional analysis to predict the potential mechanism could influence the diagnosis and prognosis.

## Methods and materials

### Date preparation

The expression of TIMP gene family, including *TIMP1*, *TIMP2*, *TIMP3* and *TIMP4*, and clinical information were acquired from TCGA (http://tcga-data.nci.nih.gov/tcga, accessed July 1st 2020). We downloaded the clinical information of 415 gastric cancer patients from UCSC Xena (http://xena.ucsc.edu/, accessed July 1st 2020), including age, gender, tumor stage, survival time and survival status. There were total of 351 cases were included for follow-up survival analysis after excluding the cases with missing medical data and 0-day survival time. For diagnostic analysis, 32 normal tissue and 375 cancer tissue expression level cases were included.

### Gene expression level and correlation analysis

The boxplots of TIMP genes were used to illustrate the expression level between gastric paracancerous tissue and GC. Both TCGA data we acquired and Metabolic gEne RApid Visualizer (MERAV, http://merav.wi.mit.edu/, accessed July 2nd 2020) 10 were used to perform boxplots.

Pearson correlation coefficient was used explain the correlation between TIMP genes in mRNA expression level, P<0.05 was considered statically significant. Those boxplots were generated by GraphPad Prism 8 software (GraphPad Software, La Jolla, CA, USA).

### Gene functional assessment

Gene ontology (GO) categories including biological process (BP), molecular function (MF), cellular component (CC) and Kyoto Encyclopedia of Genes and Genomes (KEGG) pathway were enriched by Database for Annotation, Visualization and Integrated Discovery v8.0 (DAVID, https://david.ncifcrf.gov/, accessed July 3rd 2020) 11, 12. The DAVID results with *P*<0.05 and FDR<0.25 were considered statistically significant.

### Gene co-expression and protein-protein network

Gene co-expression network was used to illustrate the co-expression and pathway connect in TIMP genes and potentially realted genes. GeneMANIA application (http://genemania.org/, accessed by July 4th, 2020) 13 in Cytoscape software was used to generate and analyze this network 13, 14.

Protein-protein interaction network for co-expression of TIMP and potential genes were assessed and generated by STRING v11.0 (https://string-db.org/) 15.

### Diagnostic value analysis

The diagnostic value of TIMP genes were assessed by receiver operating characteristic (ROC) curves. An area under the curve (AUC)>0.700 with P<0.05 was considered having diagnostic value and statistically significant. The ROC curves were generated by GraphPad Prism 8 software (GraphPad Software, La Jolla, CA, USA).

### Survival and Joint-effects survival analysis for prognosis

The expression of each gene was divided into low expression and low expression groups; age was divided into <65 and ≥65 groups; sex was divided into male and female groups; tumor stage was firstly divided into early stage (the combination of stage I, II) and advanced stage (the combination of stage III and IV) groups, secondly divided into 4 groups by each tumor stage. Median survival time (MST), overall survival (OS) and the Kaplan-Meier estimator with the log-rank test were used to assess patients' survival.

Uni-variate survival analysis with cox-regression model was used to adjust the genes' crude survival results by clinical information with statistically survival significant.

The joint-effects analysis used regrouped patients by adjusted genes. This result also adjusted by clinical characters. The log-rank P<0.05 was considered statistically significant. The K-M survival curves were generated by GraphPad Prism 8 software (GraphPad Software, La Jolla, CA, USA).

### Nomogram for prognosis prediction

A nomogram used to predict the survival of individual patient directly, including the element of age, *TIMP2*, *TIMP3*, and tumor stage. Every factor could be calculating the contribution points at the first line, and the total contribution points could reflect the time-related prognosis. The figure was generated by R version 3.6.1

### Gene function prediction by GSEA

Gene set enrichment analysis (GSEA) was used to predict the gene function compared with the hole expression matrix and low, high expression phenotype. The potential function of GO item including BP, MF and CC (c5.all.v7.1.symbols.gmt) and potential KEGG pathway (c2.kegg.v7.1.symbols.gmt) were also enriched and predicted. The results with P<0.05 and FDR<0.25 was considered statistically significant.

### Cell culture, siRNA reagents and antibodies

Human gastric cancer cell line, AGS, was obtained from Cell Bank of Chinese Academy of Sciences and was used for functional experiments. AGS cell was cultured in the Dulbecco's Modified Eagle's Medium (DMEM, Gibco, 8118131) with 10% fetal bovine serum (Gibco, 42F7180K), cultured in incubator under the circumstance of 5% CO_2_ and 37℃. siRNA was used for transfection of TIMP3 were from Hippo Biotecnology Company (Huzhou, Zhejiang Provence, China). Liposome bodies were purchased from Invitrogen (lipo2000). Antibodies for western blot were against TIMP3 (PROTEINTECH, 10858-1-AP).

### Cell transfection

Cells were divided into 4 groups for transfection: negative control (siTIMP3-NC), siTIMP3-1, siTIMP3-2 and siTIMP3-3. 75pmol plasmid and 10μL lipo2000 were cultured in 125μL medium without serum separately, after 5 minutes, two components were mixed. 4 groups were cultured for 6 hours, then washed by sterile phosphate buffered saline and cultured in the medium without serum for 48 hours in 6-well plate for observing and selecting the best transfection results. Total RNA was extracted for validating gene expression level after transfection.

### Cell proliferation, invasion and migration assay

Cell proliferation was performed by Cell Counting Kit-8 (CCK-8). After 48 hours' transfection, the siTIMP3-NC and siTIMP3-1 were transplanted to 96-well plate at 3000 cells per well. Add 1/10 unites of CCK-8 in each well and cultured under the circumstance of 5% CO_2_ and 37℃ for 2 hours in the time of culturing for 0 hour, 24 hours, 48 hours and 72 hours respectively. After that, the optical density was measured at 450nm wavelength.

Plate clone formation assay was also used for proliferation tests. After transfection for 48 hours, resuspended cells were transplanted to 6-well plate at 500 cells per well and 2mL medium were added in the wells respectively at the same time. After cultured under the circumstance at 37℃ and 5% CO_2_ for 12 days, the cells were washed, fixed and dried for observation.

Transwell assays and Wound healing assay were used to observe cell invasion and migration ability.

## Results

### Gene function and correlated assessment

**Figure 1:**
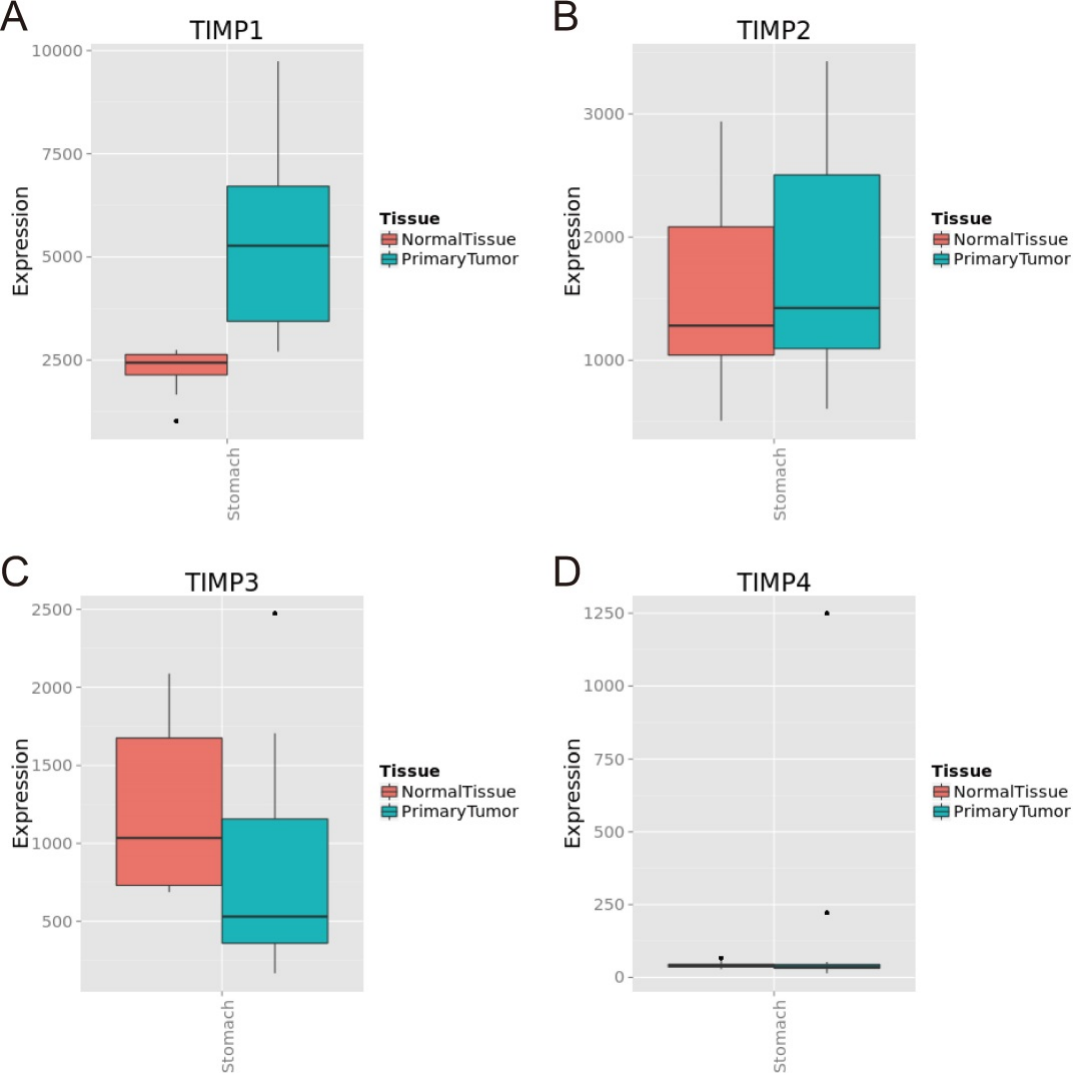
TIMP gene expression level in normal gastric tissue and tumor tissue in MERAV database. (A) TIMP1, (B) TIMP2, (C) TIMP3, (D) TIMP4.

**Figure 2:**
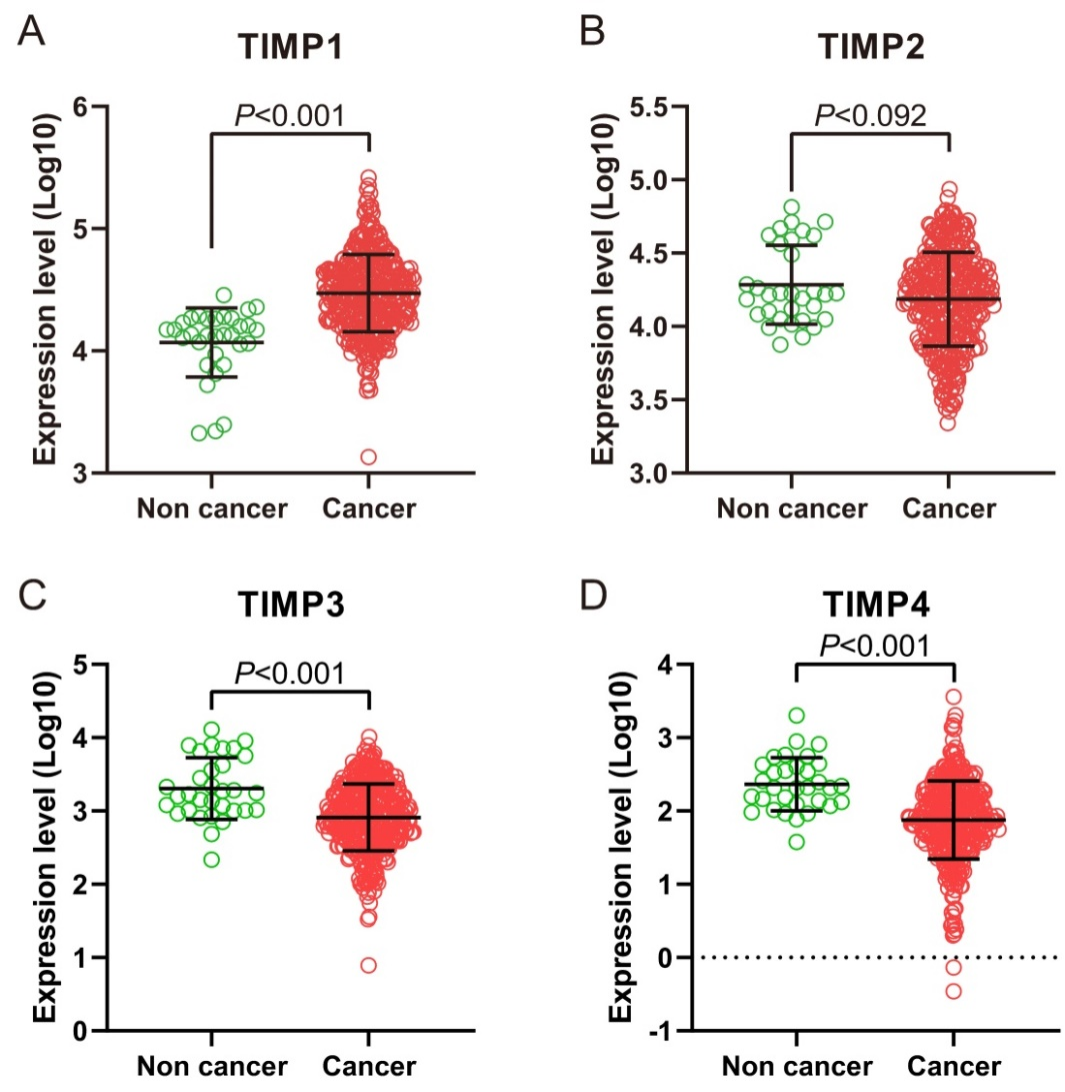
TIMP gene expression level in normal gastric tissue and tumor tissue in TCGA database. (A) TIMP1, (B) TIMP2, (C) TIMP3, (D) TIMP4.

We found *TIMP1* was expressed in GC tissue was higher than normal gastric tissue (Figure 1A and 2B), *TIMP3* and *TIMP4* were expressed in normal gastric tissue were higher than GC tissue (Figure 1C, 1D, 2C and 2D). As for *TIMP2*, there were no significance between two type tissues (*P*>0.05, Figure 2B).

**Figure 3:**
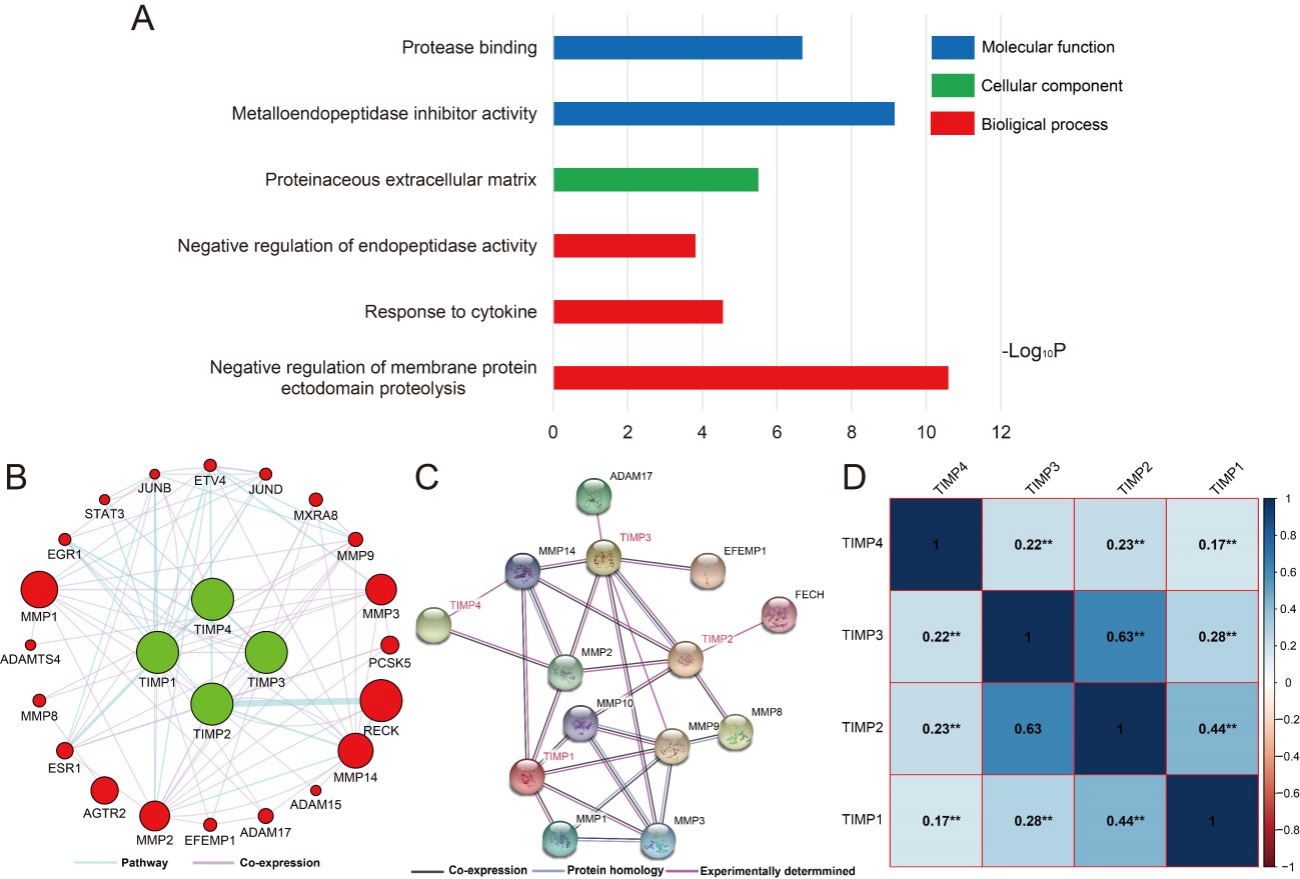
Functions and correlation of TIMP genes. (A) GO enrichment analysis by DAVID, (B) co-expression and pathway network, (C) protein-protein interaction network, (D) correlation analysis, **, P<0.001.

By gene function enrichment analysis, we verified and confirmed TIMP gene family were associated with protease binding, metallopeptidase inhibitor activity, proteinaceous extracellular matrix, negative regulation of endopeptidase activity, response to cytokine and negative regulation of membrane protein ectodomain proteolysis (Figure 3A, Supplementary Table 1). TIMP genes also have connections with *STAT3*, *MMP3*, *MMP8*, *MMP14* and other genes at pathway and co-expression level (Figure 3B and 3C). In the family, except there was no correlation significance between *TIMP2* and *TIMP3*, the rest of the combination correlated more or less (Figure 3D).

### Diagnostic value

**Figure 4:**
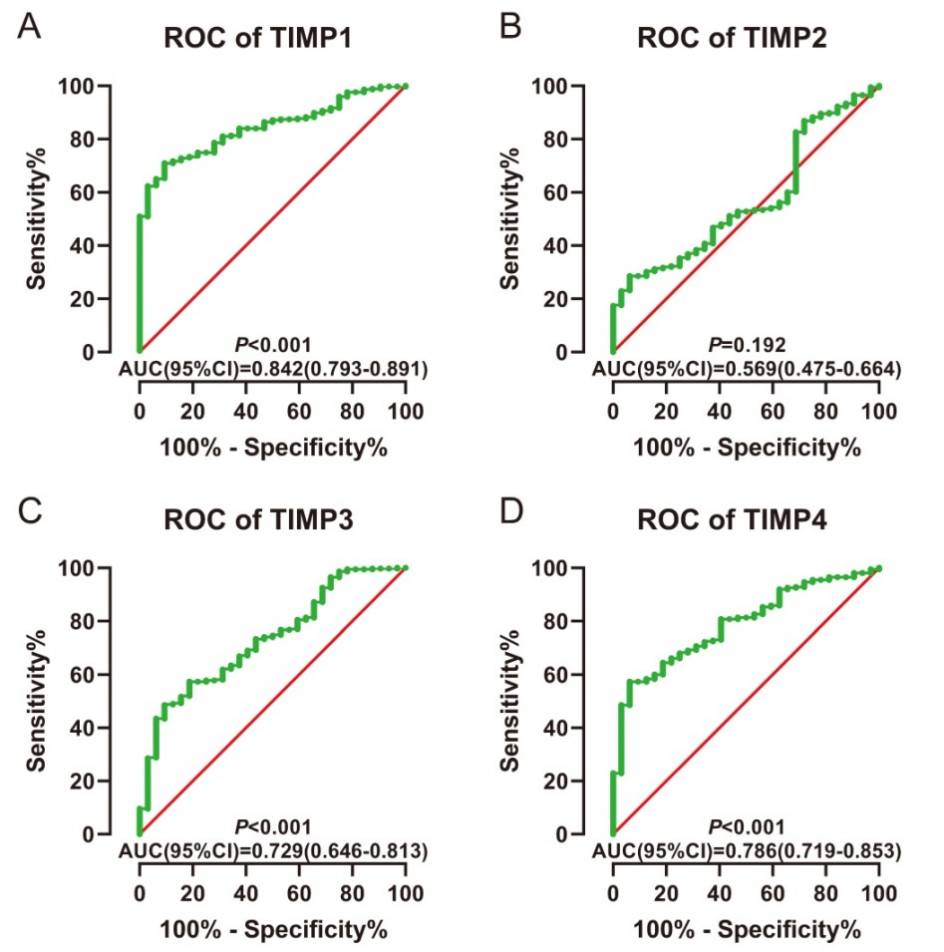
ROC for diagnosis. (A) TIMP1, (B) TIMP2, (C) TIMP3, (D) TIMP4.

The AUC was larger than 0.70 could be thought having a diagnostic value in ROC curves. *TIMP1*, *TIMP3* and *TIMP4* have diagnostic value (AUC=0.842, 0.729, 0.786; all *P*<0.001) (Figure 4A, 4C and 4D).

### Prognostic value

**Figure 5:**
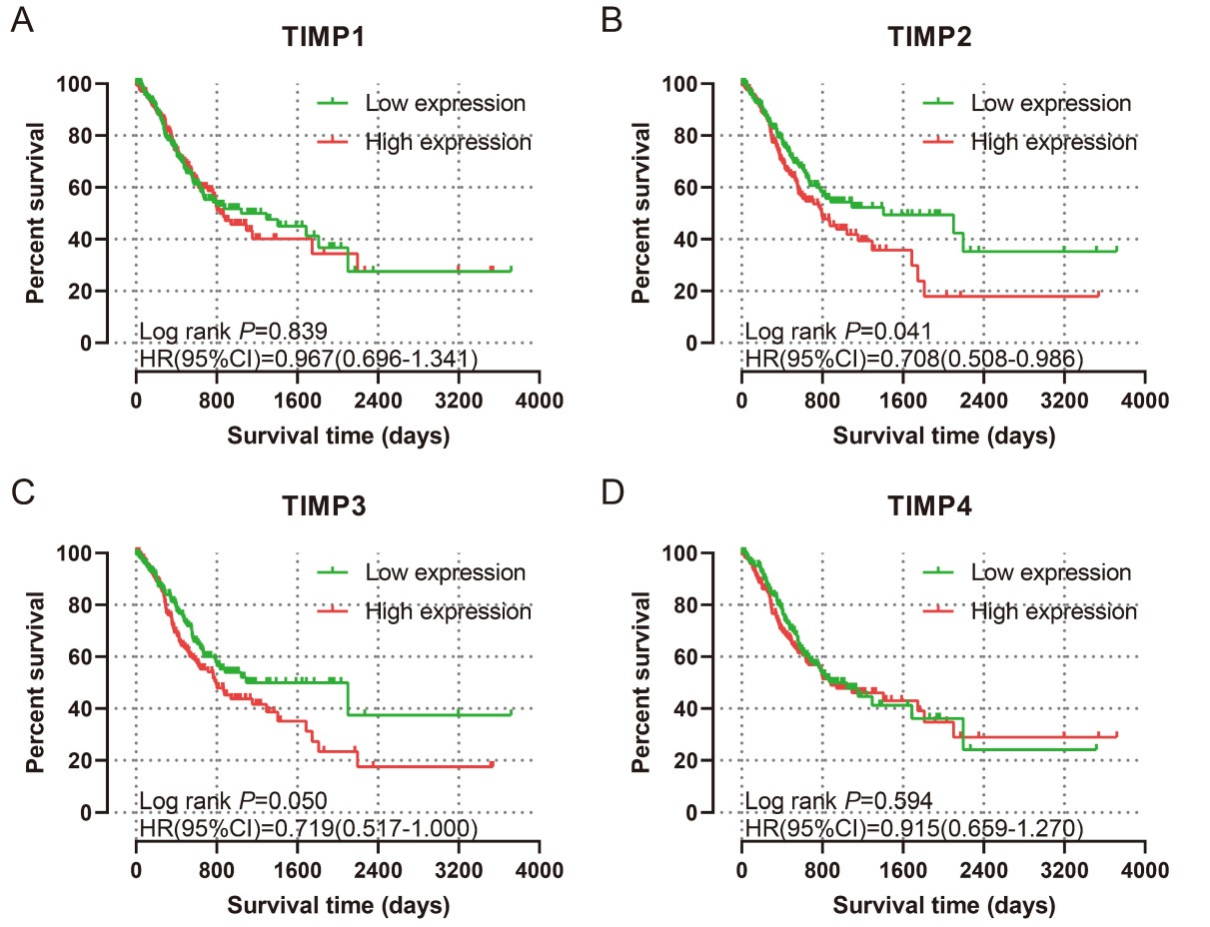
K-M plots for prognosis. (A) TIMP1, (B) TIMP2, (C) TIMP3, (D) TIMP4.

The baseline data and analysis results were shown in Table 1. Only age and tumor stage are the factors that could influence the prognosis. A for uni-variate survival analysis for genes, the expression level of *TIMP2* and *TIMP3* have prognosis value. Low expression of *TIMP2* and *TIMP3* associated with favourable OS (all *P*<0.05) (Figure 5B and 5C). Multi-variate survival analysis was performed by adjusting age and tumor stage. The results revealed that low expression of *TIMP2* and *TIMP3* associated with favorable MST (all P<0.05) (Table 2), which correspond with uni-variate survival analysis.

### Nomogram

**Figure 6:**
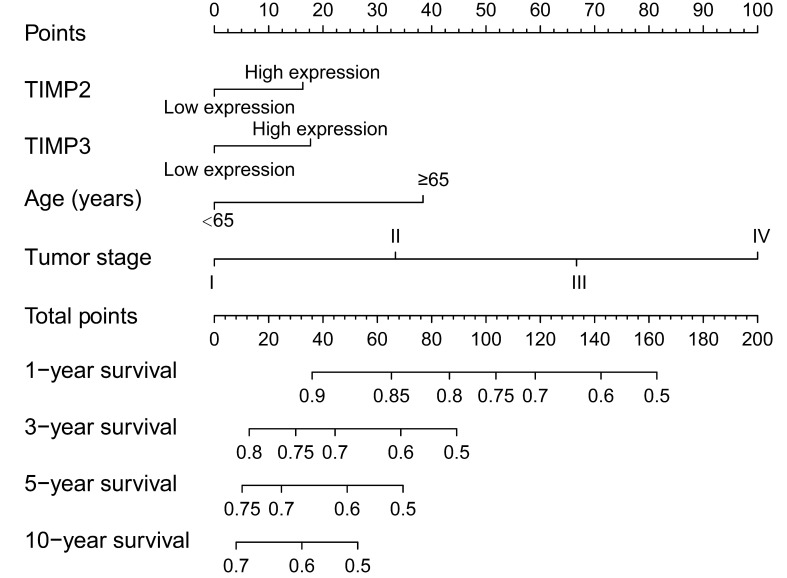
Nomogram for the predication of individual survival rate.

The contribution points of high expression of TIMP2 and TIMP3 are approximately the same, the points between 10 and 20. Contribution points of tumor stage are high, raising by the advancing tumor stage. In the nomogram, an individual patient with high total contribution points may have a worse survival rate (Figure 6).

### Joint-effects survival analysis

The patients were regrouped into 4 by the different combination of *TIMP2* and *TIMP3* expression level (Table 3). The grouping information was showen in Table 4. We found that all low expression of *TIMP2* and *TIMP3* have favorable OS and MST (Group1), compared with all high expression (Group 4) (*P*<0.05 Figure 6, Table 4).

### Potential gene function prediction

**Figure 8:**
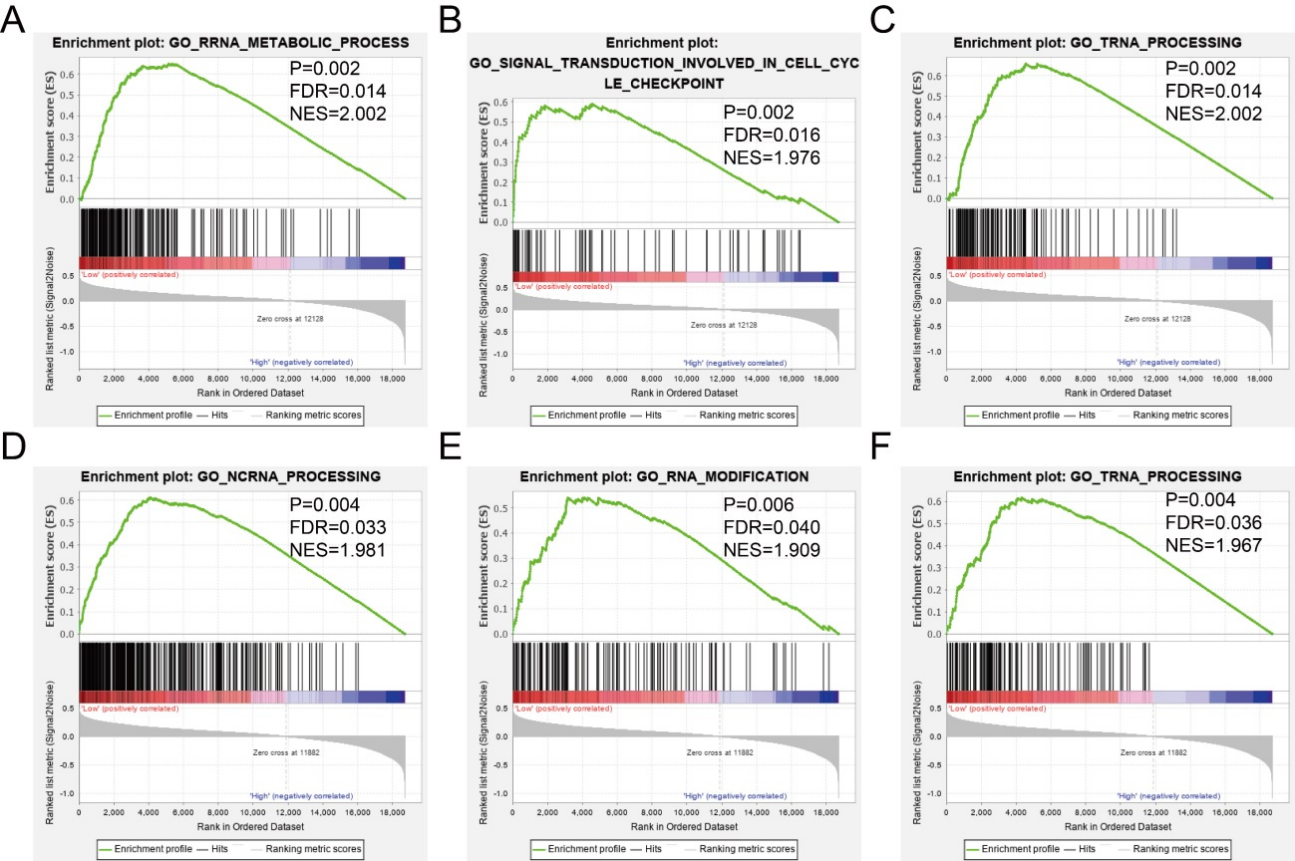
GSEA results of GO enrichment analysis. (A-C) the results for *TIMP2*, (D-F) the results for *TIMP3*.

**Figure 9:**
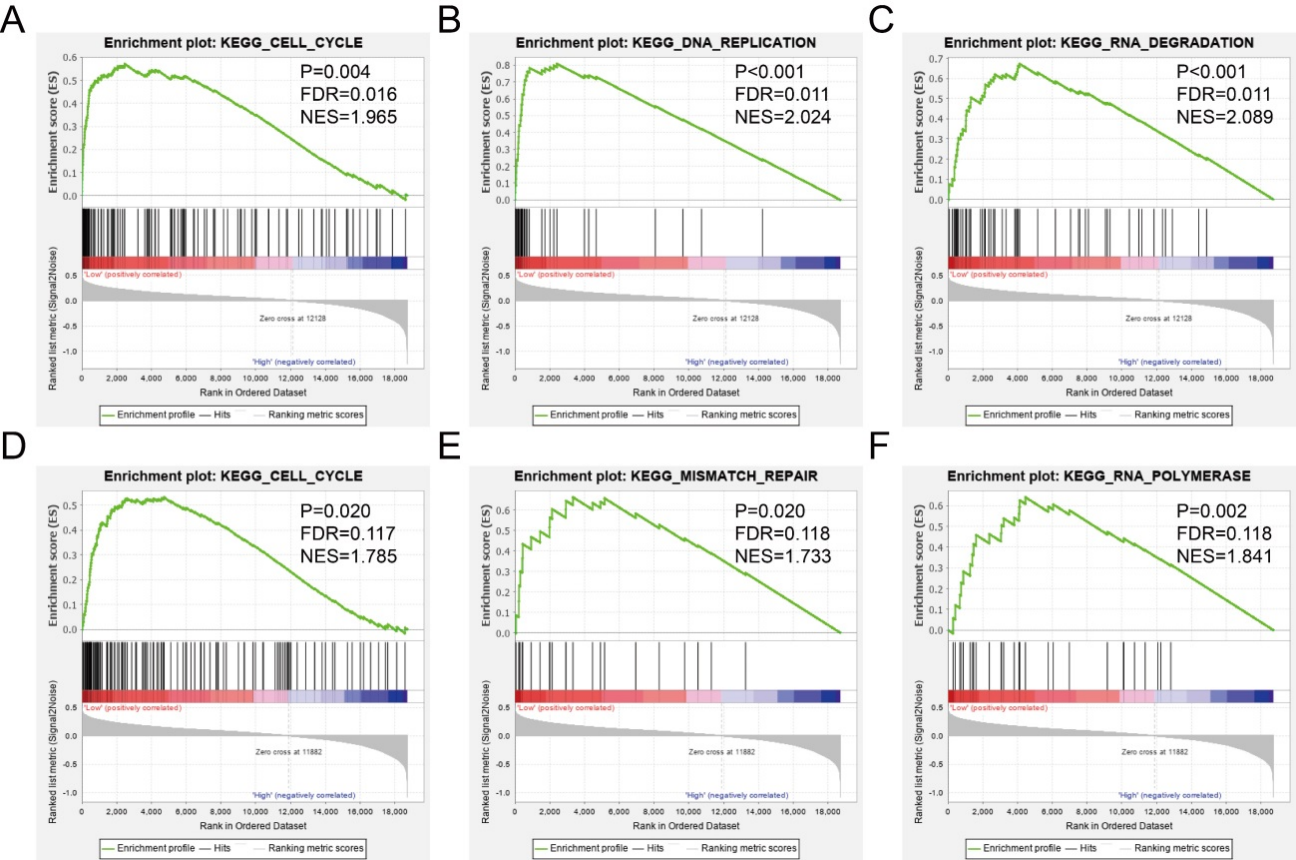
GSEA results of KEGG enrichment analysis. (A-C) the results for *TIMP2*, (D-F) the results for *TIMP3*.

The GSEA was used to predict potential gene function and associated KEGG pathway. *TIMP2* was found to be associated with RNA metabolic process (Figure 8A), signal transduction involved in cell cycle checkpoint (Figure 8B), tRNA processing (Figure 8C), cell cycle pathway (Figure 9A), DNA replication pathway (Figure 9B) and RNA degradation (Figure 9C). *TIMP3* was found to be associated with ncRNA processing (Figure 8D), RNA modification (Figure 8E), tRNA processing (Figure 8F), cell cycle pathway (Figure 9D), mismatch repair (Figure 9E) and RNA polymerase (Figure 9F). The rest of the enrichment result was shown in Supplementary Table 2 and Supplementary Table 3.

### Identification of silencing by siRNA, RT-PCR and Western Blot results

**Figure 10:**
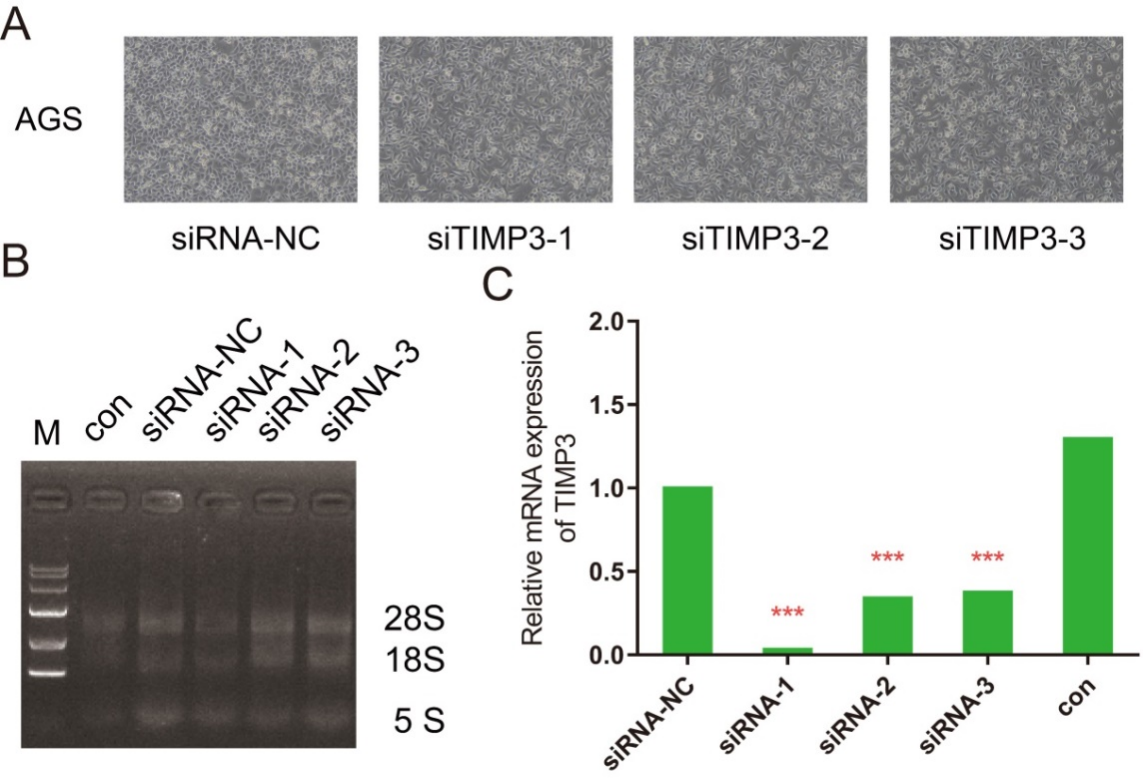
Observing the transfection results for silencing of *TIMP3*. (A) AGS cells for independent groups under microscopes, (B) RT-PCR photograph results, (C) relative mRNA expression of TIMP3 by RT-PCR.

Four groups of AGS cells after transfection were showed in Figure 10A. RNA electrophoresis pattern was showed in Figure 10B and 10C. The expression levels of TIMP3 in the siRNA-1, siRNA-2, and siRNA-3 groups were significantly down-regulated compared with NC group, moreover the result of siRNA-1 group was the best (0.062), meanwhile the siRNA-2 (0.499) and siRNA-3 (0.495) groups were down-regulated at almost the same level in AGS cell.

**Figure 11:**
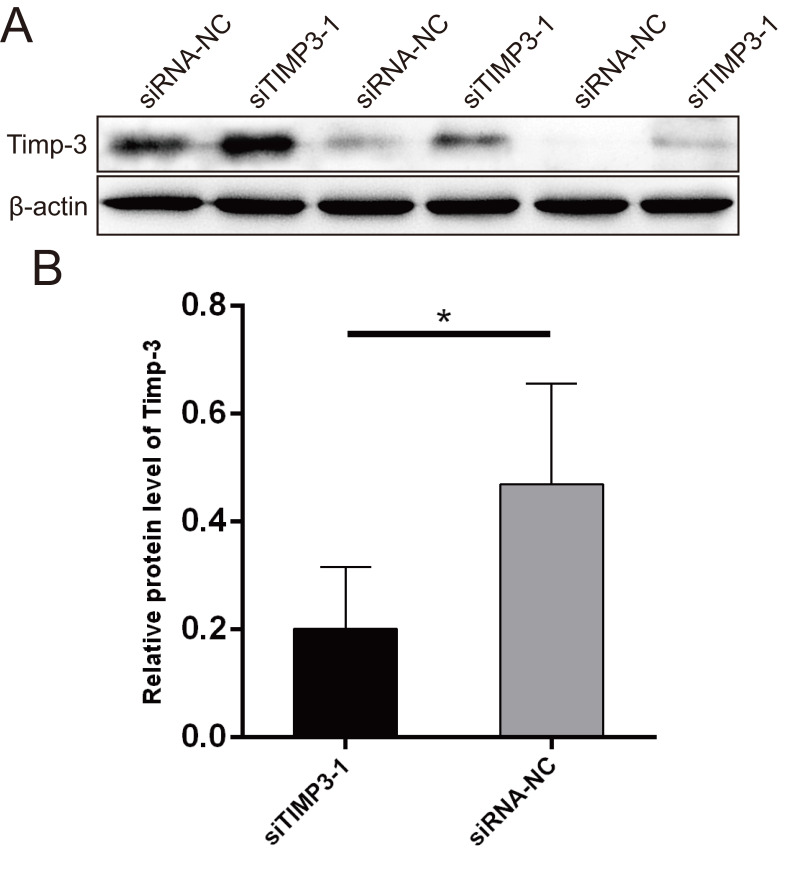
Western blots for validating the silencing of TIMP3. (A) WB photograph results, (B) relative mRNA expression of TIMP3 by WB.

The WB results showed that the Timp-3 protein expression level in the siTIMP3-1 group was significantly lower than that in the siRNA-NC group after transfection (Figure 11).

### Cell proliferation, invasion, migration and clone assays

**Figure 12:**
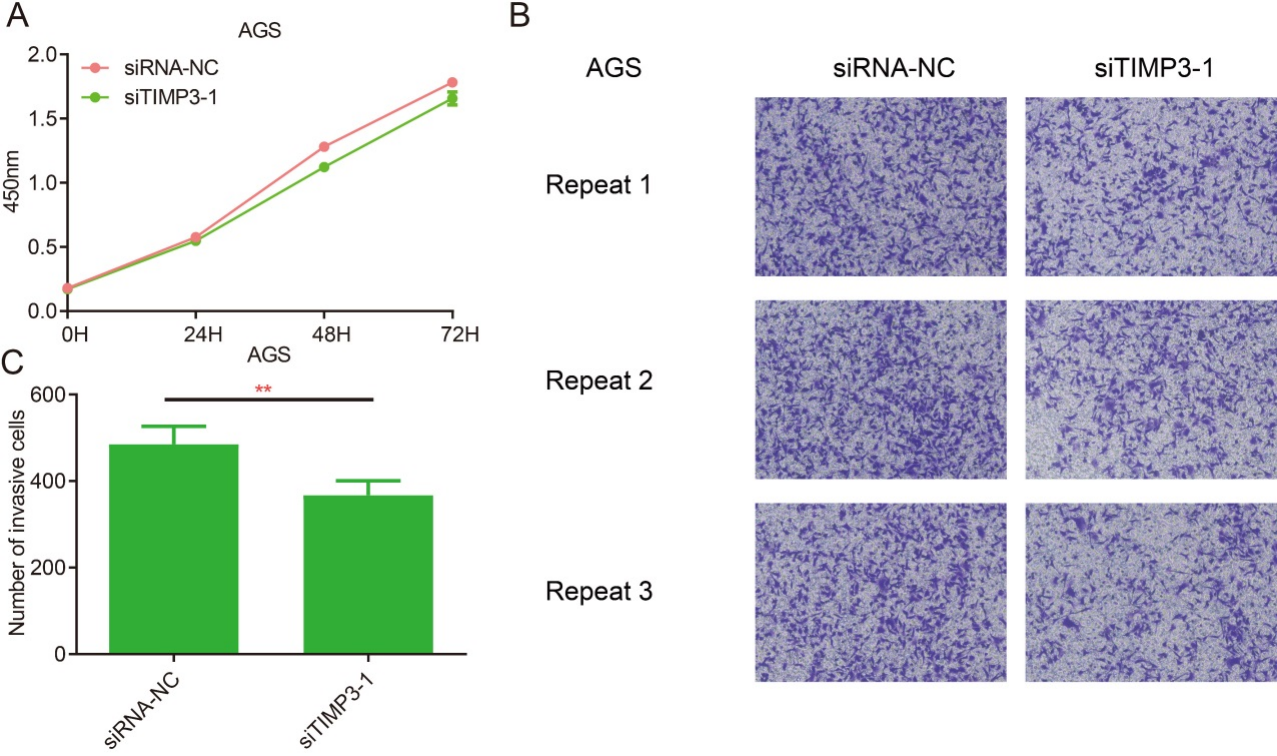
Cell proliferation and migration assays for TIMP3. (A) proliferation assays, (B) Transwell assays, (C) the analysis of Transwell results, **, P<0.05.

The results of CCK8 at each time point after transfection showed that compared with the siRNA-NC group, the proliferation ability of AGS cells in the siTIMP3-1 group had no significant change (Figure 12A).

The results of the invasion assays showed that after transfection, compared with the siRNA-NC group, the migration ability of AGS cells in the siTIMP3-1 group was significantly weakened (Figure 12B and 12C).

**Figure 13:**
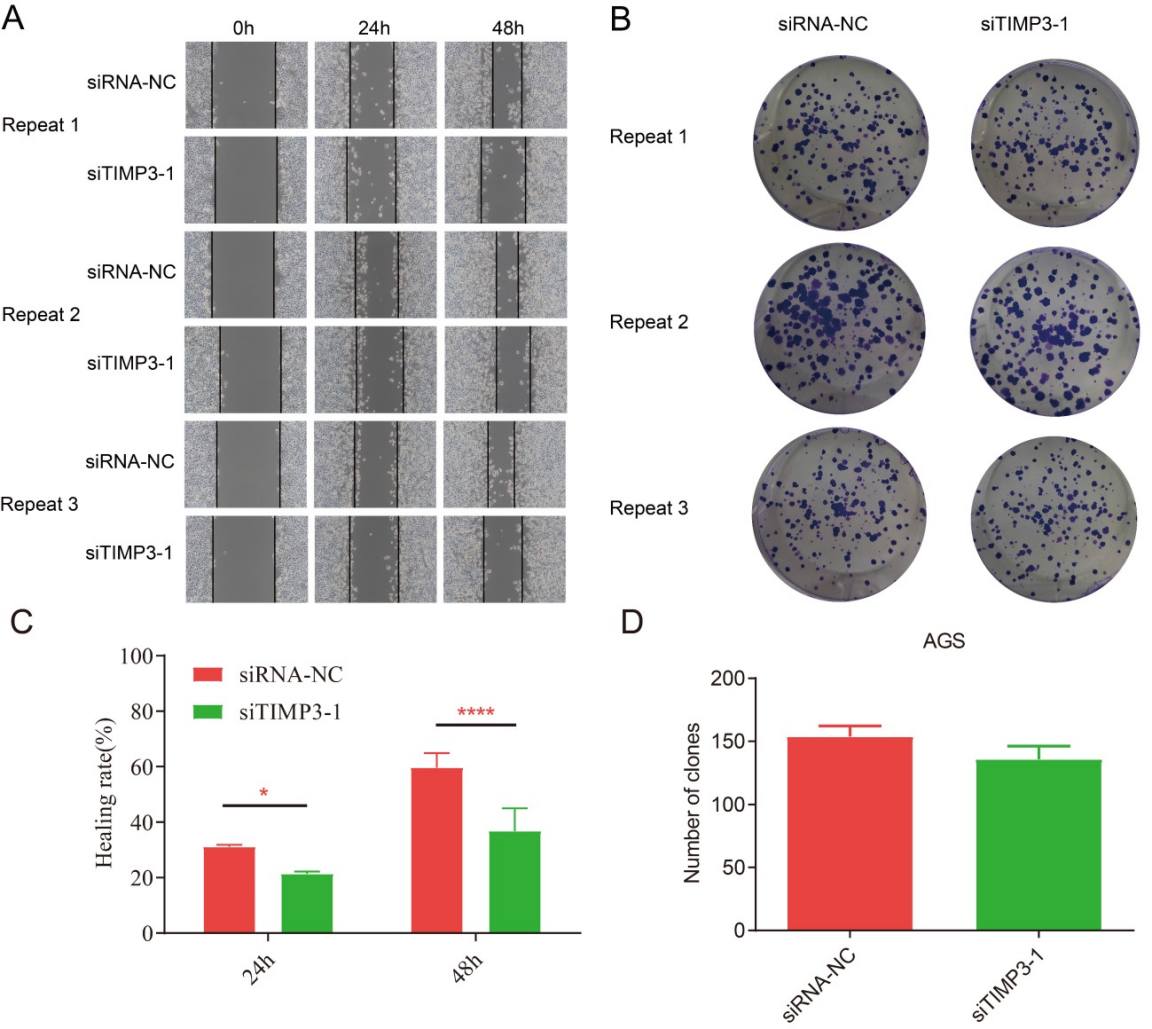
Cell migration and clone assays. (A) Cell migration assay for negative control group and silencing TIMP3 group in 0h, 24h and 28h, (B) cell clone assays for NC group and silencing TIMP3 group, (C) the compare of healing rate of migration assays, *, P<0.0025, ****, P<0.0001, (D) the compare of number of clones between NC group and silencing TIMP3 group.

The wound healing assays results of independent repeated experiments showed that after transfection, the migration rate of AGS cells in the siTIMP3-1 group was lower than that in the siRNA-NC group, and the difference was significant at 48 hours (Figure 13A and 13C).

The results of the cell clone assays showed that after transfection, compared with the siRNA-NC group, the number of AGS cell clone formation in the siTIMP3-1 group was slightly reduced, but there was no significant difference (Figure 13B and 13D).

## Discussion

TIMP genes belong to tissue inhibitor of metallopeptidases (TIMP) gene family. There are total of 4 genes in this family, including *TIMP1*, *TIMP2*, *TIMP3* and *TIMP4* 16. The most known function of *TIMPs* was regulating the balance of metabolism extracellular matrix 17, 18. We found *TIMP2* and *TIMP3* have a correlation with *matrix metalloproteinases* (*MMPs*). Also, *TIMPs* were the natural inhibitors and regulators of *MMPs* and acted as critical rules in cancer 17, 19. Until now, the report of diagnostic and prognostic value is few.

However,* TIMPs* was found to be associated with serval cancers. In human colon cancer, overexpressed of *TIMP1* associated with worse OS. Knockdown *TIMP1* could inhibit the proliferation of colon cancer cell line by regulating FAK-PI3K/AKT and MAPK pathway, however we have not found the relationship between *TIMP1* expression and patients' outcomes.

As for *TIMP2*, the former researcher found that high expression of *TIMP2* was associated worse prognosis of GC patients 20, which corresponding with our research, but the diagnostic value was not illustrated. Likewise, in ovarian cancer, depressing *TIMP2* by *EZH2* genes, aggressive behaviors like invasion and migration could be repressed 21. But in different human cancers, hepatocellular carcinoma, for instance, *TIMP2* was usually down-regulated, and associated with worse prognosis and more aggressive tumor behavior 22. This phenomenon could not only explain by the function of oncogene and tumor suppressor gene, and this may cause by the changed circumstance, the function of the gene expression level was changed in different cancer type.

*TIMP3* was reported down-regulated in GC tissue 23, unfortunately, there were few reports about the prognosis value and diagnosis value in GC. Most research about *TIMP3* in GC, focusing on methylation 23, 24. In mRNA expression research, contradict with it in other cancers 25, we found over-expressed *TIMP3* associated with worse OS, which means this phenomenon may cause by accelerating the aggressive behavior. In breast cancer mice model, deficient of *Timp3* could resist developing of breast cancer by inhibiting tumor cell's growth 26.

To explore the potential mechanism of *TIMP2* and *TIMP3* in GC prognosis, we used the GSEA to analysis. All those two genes involved in the cell cycle process and pathway, which is a critical role in cell proliferation, and effects the occurrence of cancer 27. It has been confirmed that *TIMP2* could inhibit the cell cycle process at G1 phase, by binding α3β1 integrin 28.*TIMPs* was also involved in ncRNA processing. It was reported that *TIMP2* was the direct target of miR-93 with a negative relationship in GC, contradicts with our prediction, the down-regulated of *TIMP2* associated with worse GC cell behavior 29. This former research found the gene function could not cooperate with the clinical outcome neither our study nor other studies 20, this may because *TIMP2* was not only the target by ncRNA, the function of *TIMP2* was overlapped by different genes which have not been found.* TIMP3* also involved in *DUMT3B/TIMP3/STAT1/FOXO1* pathway in breast cancer, regulated by miR-29c, knocking down of miR-29c could increase TIMP3 protein level but decrease the methylation of *TIMP3*, which promoted cell migration, invasion and proliferation 30. We could infer that a high level of *TIMP3* in breast cancer could inhibit the aggressive behavior. But in prostate cancer, *TIMP3* involved in ncRNA process accelerating the aggressive behavior 31, 32. We found that silencing TIMP3 could significantly inhibit the migration and invasion of AGS cells, but it was no use for cell proliferation and clone.

The function of diagnosis and prognosis for *TIMP2* and *TIMP3* could be affected by serval aspects including their function, ncRNA process, gene-gene correlation, but those two gene expression levels are confirmed in GC, and have a statistically significant difference. Those could be used as biomarkers, but the potential function still needs to study by some methods, including building a ceRNA regulation network, and further basic research.

Although significant results have been found, there are still some shortages in this study. First, the clinical information is not enough to include all potential high-risk factors, including alcohol history and the treatment statue such as the history of radiotherapy, chemotherapy and surgery. Second, there only one database and our research center also do not have enough cases, lacking of the independent cohort to validate the results. Third, the potential function predicted by GSEA need a basic experiment to verify.

## Conclusion

This present research is compensation of former study, we combined *TIMP2* and *TIMP3* then made a survival analysis, and found all low expression of those two genes patients have favorable prognosis, and the diagnostic value is significant. *TIMP1* and *TIMP2* could be used as diagnostic biomarkers. *TIMP2* and *TIMP3* could be used as potential biomarkers for GC's prognosis. The nomogram was built to assess survival rate for individual GC patients more directly. The silencing of TIMP3 could inhibit the migration and invasion of gastric cancer cell. The GSEA results could instruct the future basis experiment to verify those results.

## Supplementary Material

Supplementary table 1.

Supplementary table 2.

Supplementary table 3.

## Figures and Tables

**Figure 7:**
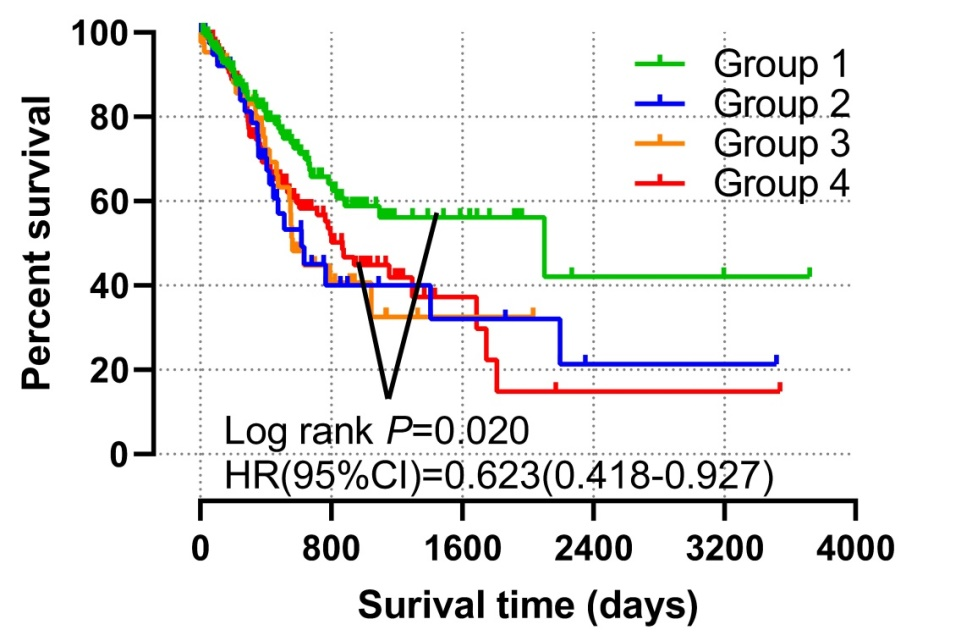
Joint-effects analysis combined by the expression level of *TIMP2* and *TIMP3*.

**Table 1:** Clinical data characteristic of 351 GC patients in TCGA.

Items	Cases (Total n=351)	No. of events (%)	MST (days)	Crude *P*	Crude HR (95%CI)
**Age**					
≥65	148	50 (33.8%)	1811	0.018	Ref.
<65	203	94 (46.3%)	799		0.660 (0.468-0.931)
**Sex**					
Male	226	100 (45.2%)	869	0.186	Ref.
Female	125	44 (35.2%)	1043		0.787 (0.552-1.122)
**Tumor stage**					
I	47	11 (23.4%)	2197	<0.001	0.260 (0.126-0.537)
II	109	34 (31.2%)	1686		0.424 (0.247-0.728)
III	147	69 (46.9%)	779		0.643 (0.397-1.042)
IV	35	22 (62.9%)	476		Ref.
missing	13				
Tumor stage*					
Early stage	156	45 (28.8%)	1811	<0.001	0.523 (0.366-0.747)
Advanced stage	182	91 (50.0%)	669		Ref.
missing	13				

Abbreviations: TNM stage, Tumor, node and metastasis stage; MST, Median survival time; Ref, Reference; HR, Hazard ratio; 95% CI, 95% Confidence interval. Note: *, early stage was combined by stage I and II, advanced stage was combined by stage III and IV.

**Table 2:** Univariate and multivariate survival analysis of TIMP gene family.

Items	Cases (Total n=351)	No. of events (%)	MST (days)	Crude *P*	Crude HR (95%CI)	Adjusted *P*	Adjusted HR (95%CI)
**TIMP1**							
Low	175	71 (40.6%)	1043	0.839	0.967 (0.696-1.341)	0.816	0.960 (0.684-1.348)
High	176	73 (41.5%)	832		Ref.		Ref.
**TIMP2**							
Low	175	62 (35.4%)	1407	**0.041**	0.708 (0.508-0.986)	**0.031**	0.685 (0.486-0.966)
High	176	82 (46.6%)	794		Ref.		Ref.
**TIMP3**							
Low	175	63 (34.0%)	1095	**0.049**	0.680 (0.484-0.955)	**0.026**	0.680 (0.484-0.955)
High	176	81 (46.0%)	794		Ref.		Ref.
**TIMP4**							
Low	175	67 (38.3%)	874	0.594	0.915 (0.659-1.270)	0.876	0.974 (0.694-1.356)
High	176	77 (33.7%)	881		Ref.		Ref.

Abbreviations: MST, Median survival time; Ref, Reference; HR, Hazard ratio; 95% CI, 95% Confidence interval; Notes: Adjusted P, Adjustment by age and tumor stage; Bold number, statistically significance.

**Table 3:** Grouping information of Joint-effect analysis.

Group	Gene expression level	
	TIMP2	TIMP3
1	Low expression	Low expression
2	Low expression	High expression
3	High expression	Low expression
4	High expression	High expression

**Table 4:** Joint-effects analysis of the combination of TIMP2 and TIMP3.

Group	Cases (Total n=351)	No. of events (%)	MST (days)	Crude *P*	Crude HR (95%CI)	Adjusted *P*	Adjusted HR (95%CI)
Group1	132	41 (31.1%)	2100	**0.002**	0.623 (0.418-0.927)	0.020	0.612 (0.405-0.925)
Group2	43	21 (48.8%)	618	0.720	1.096 (0.664-1.810)	0.432	1.227 (0.737-2.042)
Group3	43	22 (31.2%)	562	0.688	1.113 (0.682-1.814)	0.688	1.109 (0.699-1.841)
Group4	133	60 (45.1%)	869	Ref.	Ref.	Ref.	Ref.

Abbreviations: MST, Median survival time; Ref, Reference; HR, Hazard ratio; 95% CI, 95% Confidence interval. Notes: Adjusted P, Adjustment by age and tumor stage.
